# Christmas under Difficulties at Chichester

**Published:** 1913-01-04

**Authors:** 


### CHRISTMAS UNDER DIFFICULTIES AT CHICHESTER.

The Christmas festivities at the Chichester Infirmary for 1912 had to be somewhat curtailed on account of the restricted accommodation for patients and staff consequent upon the development of the reconstruction scheme in memory of King Edward VII. The number of beds available for in-patients is now only twenty-five, instead of the usual sixty, and only the most urgent cases are admitted. Nevertheless, no effort was spared by the staff to make the festival as cheerful as possible. On Christmas Eve, as in former years, the nurses, led by the matron, made a round of the wards in procession singing carols; this was, as usual, much appreciated by the patients, and was a great pleasure to the matron and nurses. On Christmas Day the nursing staff had a special breakfast laid in the Board-room, the usual dining-room being in the builders' hands. The patients received special attention on this day, special Christmas fare being provided. The turkeys and plum-pudding were carved in the wards by the house surgeon and matron, who were assisted in the serving by the Secretary, Mr. Tom B. Harrison, and by the nursing staff. Those poor mortals whose diagnosis would not permit of them being allowed to consume any of the "extra" diet eyed with envy those who were more fortunate. A distribution of fruit and the inevitable crackers followed, and by the number of adorned scalps visible no doubt existed as to the thoroughness of the enjoyment. After the patients came the domestic staff, who, as is customary, partook of their special fare in the main kitchen. Here the principal members of the resident staff forsook for a time their usual routine, and took upon themselves the duty of waiting upon those who so often wait upon them. This diversion caused much merriment, and the domestic staff had a real good time. Patients' visitors were allowed in the afternoon, and those who were able partook of a homely cup of tea with those who are near and dear to them. A special service was held in the chapel at 4 P.M., the service being conducted by the Rev. E. H. Nash, the chaplain, and as many of the patients and staff who were able attended. On Boxing Day the annual dinner for the administrative and resident staff was held, and a most convivial time was spent; toasts with full musical honours were frequently given after full justice had been done to the special fare. On Friday the usual Christmas tree was held in the Nightingale Wing, a huge tree, 16 feet high, being kindly given for the occasion by a generous donor. The tree was beautifully illuminated by electricity with coloured lamps, and this combined with the decorations had a lovely effect. Old patients and friends of the patients and the whole of the staff gathered round to witness the distribution of the presents by Santa Claus, the house surgeon, who was assisted by the Secretary and two members of the board of management. A musical programme, contributed by members of the staff, was afterwards much enjoyed.

On New Year's Day a concert was given for the benefit of the patients and staff in the Nightingale Ward by members of the Cathedral Choir, and supplemented with illusions by a local magician. The Christmas of 1912 at the Chichester Infirmary, by confession of all those who were within its walls, and in spite of the difficulties of rebuilding, has been a most enjoyable experience.

